# Association between posttest dexamethasone and cortisol concentrations in the 1 mg overnight dexamethasone suppression test

**DOI:** 10.1530/EC-12-0047

**Published:** 2012-08-30

**Authors:** Bjørn O Åsvold, Valdemar Grill, Ketil Thorstensen, Marit R Bjørgaas

**Affiliations:** 1 Department of Endocrinology St Olavs Hospital, Trondheim University Hospital P O Box 3250 Sluppen, N-7006, Trondheim Norway; 2 Department of Public Health Norwegian University of Science and Technology N-7491, Trondheim Norway; 3 Department of Cancer Research and Molecular Medicine Norwegian University of Science and Technology N-7491, Trondheim Norway; 4 Department of Medical Biochemistry St Olavs Hospital, Trondheim University Hospital N-7006, Trondheim Norway

**Keywords:** Cushing's syndrome, dexamethasone suppression test, cortisol, dexamethasone

## Abstract

It has been suggested that comparison of posttest dexamethasone and cortisol concentrations may improve the evaluation of the dexamethasone suppression test (DST) for Cushing's syndrome. In particular, this would be reasonable if posttest cortisol differs by dexamethasone levels within the range that is usually attained in the DST. Using fractional polynomial regression, we therefore studied the association between posttest 0800 h dexamethasone and cortisol levels in 53 subjects without Cushing's syndrome who were tested with the 1 mg overnight DST. Plasma dexamethasone was associated with plasma cortisol (*P*<0.001), and the regression line suggested a strong negative association related to dexamethasone levels <5 nmol/l. However, among the 94% of subjects with plasma dexamethasone >5.0 nmol/l, there was no association between dexamethasone and cortisol levels (*P*=0.55). In conclusion, subjects tested with the 1 mg overnight DST usually attain an 0800 h plasma dexamethasone >5 nmol/l, and plasma cortisol does not differ by plasma dexamethasone in these subjects. This suggests that routine comparison of dexamethasone and cortisol levels may not be a useful approach to improve the performance of the 1 mg DST. However, dexamethasone measurements may identify subjects with inadequately low plasma dexamethasone and may therefore be of value when retesting subjects with possibly false-positive DST results.

## Introduction

The 1 mg overnight dexamethasone suppression test (DST) is a common initial test for endogenous Cushing's syndrome (1). The principle of the test is that dexamethasone will suppress ACTH and cortisol secretion in healthy individuals but not in people with Cushing's syndrome. Sometimes, however, dexamethasone may be poorly absorbed or excessively metabolized. As a result, the dexamethasone concentration attained after intake of 1 mg dexamethasone may be too low to suppress pituitary ACTH secretion, causing a false-positive test result (1). Therefore, it has been suggested that comparison of posttest dexamethasone and cortisol, e.g. by calculating an index of the two concentrations, may improve the evaluation of the DST (2, 3, 4, 5). This suggestion would be particularly reasonable if posttest cortisol differs by dexamethasone levels within the range that is usually attained in the DST. Therefore, we have studied the association between dexamethasone and cortisol concentrations in subjects without Cushing's syndrome who were tested with the 1 mg overnight DST.

## Materials and methods

### Subjects

At the Department of Endocrinology, Trondheim University Hospital, we studied consecutive patients who were tested with 1 mg DST on suspicion of endogenous Cushing's syndrome between February 2008 and March 2010. Subjects were instructed to take two tablets of 0.5 mg dexamethasone at 2300 h. The following morning, before eating and tooth brushing, they collected a salivary sample by placing a cotton swab (Salivette; Sarstedt, Nümbrecht, Germany) between the cheek and lower teeth for at least 1 min. The cotton swab was delivered at our hospital's Department of Medical Biochemistry the same morning at 0800 h. At the same time, blood was drawn for cortisol and dexamethasone measurements.

Fifty-nine subjects provided blood for cortisol measurement, and from 56 of these subjects, plasma was stored at −70 °C for future analysis of dexamethasone. The subsequent clinical evaluation was performed without knowledge of dexamethasone levels. The DST was considered normal if posttest plasma cortisol was <100 nmol/l. Fourteen of the 56 subjects had posttest plasma cortisol >100 nmol/l, and three of them were subsequently diagnosed with Cushing's syndrome or lost to follow-up. In the remaining 11 subjects, the suspicion of Cushing's syndrome was refuted after a clinical endocrinological evaluation including normal results of additional biochemical testing (repeated DST, midnight salivary cortisol, or diurnal variation in plasma cortisol; *n*=9), or after an endocrinological evaluation without additional tests for Cushing's syndrome (*n*=2). Among subjects with normal DST (defined as posttest plasma cortisol <100 nmol/l), 12 had posttest salivary cortisol higher than the suggested cutoff of 3.7 nmol/l (6). However, in these subjects, the suspicion of Cushing's syndrome was refuted for the following reasons: posttest plasma cortisol <50 nmol/l (*n*=7), endocrinological evaluation including normal results of additional tests for Cushing's syndrome (*n*=4), and one subject with adrenocortical adenoma was diagnosed with primary hyperaldosteronism, which was normalized after adrenalectomy. Thus, 53 subjects without Cushing's syndrome were included in the analyses.

### Laboratory measurements

The samples were analyzed at the Department of Medical Biochemistry, Trondheim University Hospital. Plasma and salivary cortisol were measured in the fresh samples using an immunological method on a Roche Modular E (plasma: reference range for morning samples 142–651 nmol/l, analytical variation 6.6% at 276 nmol/l and total variation 21.9%; saliva: reference range for morning samples 6–29 nmol/l, analytical variation 7.9% at 12 nmol/l). Samples for posttest salivary cortisol measurement were available from 37 of the 53 subjects. Six subjects did not provide a salivary sample, and ten cotton swabs contained too little saliva. In eight of the 37 salivary samples, cortisol was below the minimal detection limit of 1 nmol/l, and these were assigned the half value of the minimal detection limit.

Plasma dexamethasone was measured during March 2010 using the direct RIA from IgG Corporation (Nashville, TN, USA) as described by Ritchie *et al*. (7, 8), with minor modifications. The tracer was [1,2,4,6,7-^3^H]-dexamethasone, 54 Ci/mmol (NET11920001MC, Perkin Elmer Life and Analytical Sciences, Waltham, MA, USA) evaporated to dryness under nitrogen and diluted to ∼80 000 d.p.m./ml in RIA buffer (0.063 mol/l Na_2_HPO_4_, 0.013 mol/l Na-EDTA, 0.2 g/l NaN_3_, 1 g/l bovine gamma globulin, and 0.01 g/l 8-anilino-1-naphthalene sulfonic acid, pH 7.4). Standard was Dexamethasone Vetranal, analytical standard (Sigma–Aldrich), diluted in absolute ethanol. Standards spanning the concentration range 0.41–25.5 nmol/l were prepared fresh for each run by dilution in RIA buffer. A 6.4 nmol/l control sample was prepared by adding dexamethasone standard to dexamethasone free plasma. All samples were assayed in duplicate and counted in a Tri-Carb 2900TR liquid scintillation analyzer (Perkin Elmer Life and Analytical Sciences, Shelton, CT, USA) with automatic quench correction.

### Statistical analyses

We used a scatter plot to assess the association between posttest plasma dexamethasone and cortisol. We aimed to examine whether there was a cutoff for plasma dexamethasone below which cortisol was not suppressed, and the scatter plot suggested the presence of such a cutoff around 5 nmol/l (to convert to μg/liter, divide by 2.548). Subsequently, we used fractional polynomial regression to assess the association between dexamethasone and cortisol levels, both across the entire dexamethasone range and separately among people with dexamethasone >5.0 nmol/l. We only allowed one power term in the fractional polynomial regression model; allowing two power terms did not substantially improve the model. Cortisol levels were log-transformed due to non-normal distribution.

A pretest morning plasma cortisol measurement was available in 48 of the 53 subjects. In similar analyses, we assessed the association of dexamethasone with the log-transformed ratio between post- and pretest cortisol levels. The association between dexamethasone and posttest salivary cortisol levels was assessed using Kendall's rank correlation coefficient. Among 45 subjects for whom body mass index (BMI; weight in kg divided by the squared value of height in meters) was available, we examined the association of BMI with plasma dexamethasone using linear regression and studied whether inclusion of BMI in the model influenced the association between dexamethasone and cortisol levels. Additionally, we stratified all analyses by the reason for DST (clinical suspicion of Cushing's syndrome (*n*=27) or evaluation for adrenal incidentaloma (*n*=26)).

In two subjects with positive DST, the suspicion of Cushing's syndrome was refuted by clinical evaluation without further tests for Cushing's syndrome, and in these subjects, subclinical Cushing's syndrome cannot be excluded. We therefore repeated the analyses, omitting these two subjects, as well as the three subjects with previous treatment for Cushing's disease. The data were analyzed using Stata version 10.1 for Windows (Stata Corp., College Station, TX, USA). The samples were collected from subjects who were tested with DST as part of routine diagnostics, and the study was exempted from ethical review by the regional medical ethics committee and approved by the privacy ombudsman (Norwegian Social Science Data Services) without the need for informed consent.

## Results

Characteristics of the subjects are shown in Table 1. Median posttest cortisol levels were 48 nmol/l (interquartile range (IQR) 28–81) in plasma and 4 nmol/l (IQR 1–9) in saliva, and median ratio of post- to pretest plasma cortisol was 0.09 (IQR 0.05–0.18). Mean plasma dexamethasone was 11.7 nmol/l (s.d. 5.9; median 10.2, range 0.8–25.7). Posttest plasma cortisol was >100 nmol/l in 11 (21%) subjects and >50 nmol/l in 25 (47%) subjects. One subject used medication (efavirenz) that may induce CYP3A4 and thereby accelerate dexamethasone metabolism; in this subject, plasma dexamethasone was 2.3 nmol/l, and posttest cortisol was 408 nmol/l in plasma and 10 nmol/l in saliva. None of the women reported use of the contraceptive pill.

**Figure 1:**
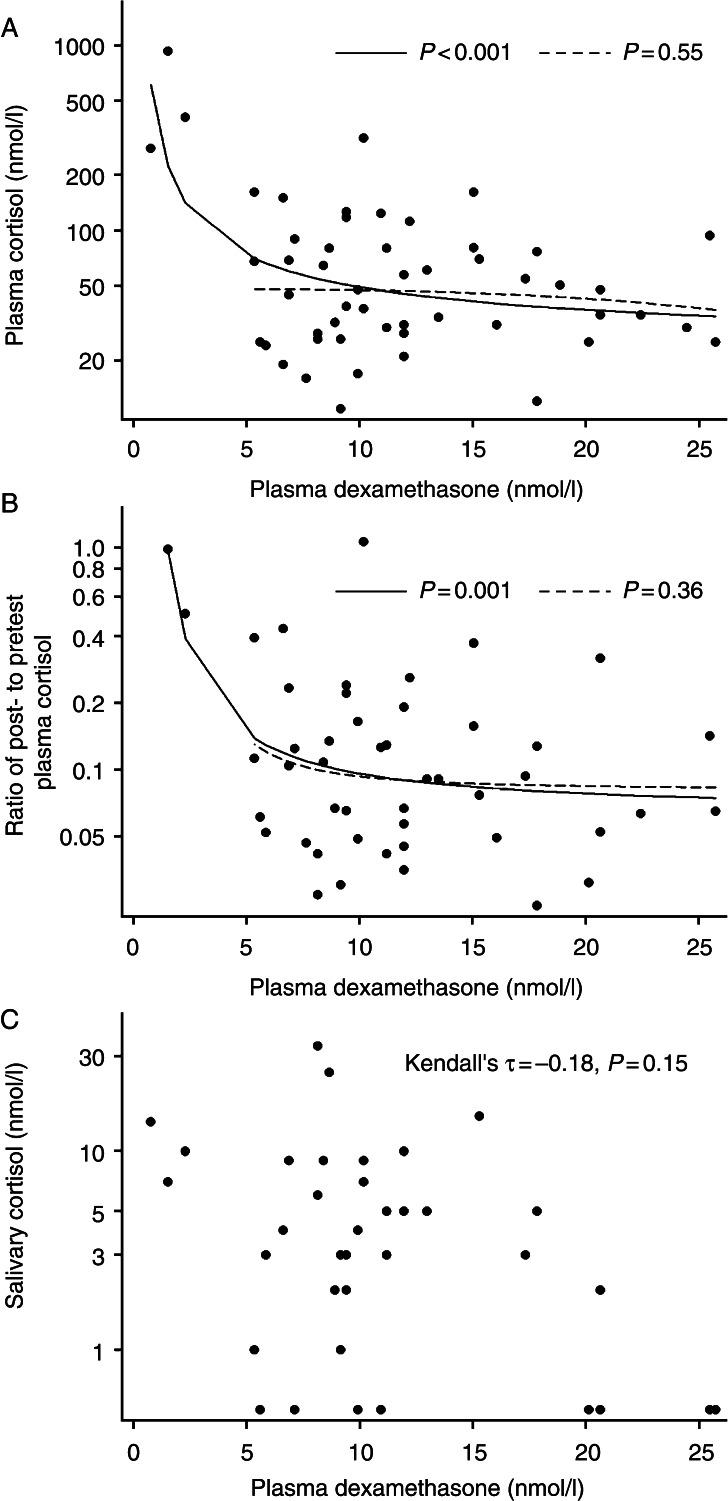
The association of posttest 0800 h plasma dexamethasone with posttest 0800 h plasma cortisol (*n*=53; A), ratio of post- to pretest morning plasma cortisol (*n*=48; B), and posttest morning salivary cortisol (*n*=37; C) among subjects without Cushing's syndrome who underwent the 1 mg overnight dexamethasone suppression test. The fractional polynomial regression lines display the associations across the entire plasma dexamethasone range (solid lines) and among subjects with dexamethasone >5.0 nmol/l (dashed lines).

Three subjects had plasma dexamethasone <5 nmol/l, and cortisol was not suppressed in any of them. Among the 50 (94%) subjects with dexamethasone >5.0 nmol/l, the scatter plot indicated no association between plasma dexamethasone and cortisol levels. In the fractional polynomial regression analysis, dexamethasone levels were associated with posttest plasma cortisol (*P*<0.001, Fig. 1A), and the regression line suggested a strong negative association related to dexamethasone <5 nmol/l. Among subjects with dexamethasone >5.0 nmol/l, however, there was no association between dexamethasone and cortisol levels (*P*=0.55, Fig. 1A). The association of dexamethasone levels with the ratio of post- to pretest plasma cortisol displayed essentially similar pattern as the association of dexamethasone with posttest plasma cortisol (*P*=0.001 across the entire dexamethasone range and *P*=0.36 among subjects with dexamethasone >5.0 nmol/l, Fig. 1B).

Dexamethasone levels were not significantly associated with posttest salivary cortisol (Kendall's *τ*=−0.18, *P*=0.15). Also, the scatter plot did not suggest any strong association between dexamethasone and salivary cortisol levels (Fig. 1C).

BMI was not associated with plasma dexamethasone (*P*=0.41). Inclusion of BMI in the model did not substantially influence the association between plasma dexamethasone and cortisol.

The association of dexamethasone with cortisol levels did not substantially differ between subjects with clinically suspected Cushing's syndrome and subjects who underwent DST because of adrenal incidentaloma. The association remained essentially unchanged after adjustment for time of blood sampling and after exclusion of two subjects with blood sampled later than 0900 h. Further, the results were essentially unchanged after omitting three subjects with previous treatment for Cushing's disease, and two subjects in whom subclinical Cushing's syndrome could not be excluded.

## Discussion

In this study of the 1 mg overnight DST, 94% of the subjects attained an 0800 h plasma dexamethasone >5.0 nmol/l, and in these subjects, dexamethasone levels were not associated with posttest cortisol levels. In subjects with dexamethasone <5 nmol/l, cortisol was not suppressed.

False-positive results of the 1 mg overnight DST are quite common. In previous studies, the specificity of the test was around 90% with plasma cortisol cutoff at ∼100 nmol/l (9). Using the recently suggested cutoff at 50 nmol/l (1), the specificity was 80% in a study of Cushing's syndrome suspects (10) but only 58% in a study of pseudo-Cushing's syndrome (11). Reasons for false-positive DST results include reduced absorption of dexamethasone and the use of alcohol or drugs that accelerate dexamethasone metabolism by inducing CYP3A4. Conversely, liver failure, renal failure, and the use of drugs that inhibit CYP3A4 may impair dexamethasone clearance (1). Thus, the attained dexamethasone level following peroral administration varies substantially between individuals, which gives the rationale for suggesting dexamethasone measurements as part of the DST.

In 1975, Meikle *et al*. (3) described a reciprocal association between dexamethasone and cortisol levels in the DST. This association has received little attention in the endocrinological literature since Meikle's studies (2, 3), but studies of the DST in psychiatric settings have confirmed a negative association between dexamethasone and cortisol levels (5, 7, 12, 13). Some authors have suggested that the performance of the DST may be improved by calculating an index of cortisol and dexamethasone levels (4, 5). Others have suggested that the DST performs well only within a certain range of dexamethasone levels and that the association between dexamethasone levels within this range and posttest cortisol is weak or absent (7, 12).

Our finding of no association between dexamethasone levels within the expected range and posttest cortisol suggests that routine comparison of these measures may not be a useful approach to improve the performance of the DST. However, dexamethasone measurements may be appropriate when retesting patients with positive DST results. In such patients, identifying an inadequately low dexamethasone level may provide an explanation for the positive DST result (2). Plasma dexamethasone of 5.6 nmol/l has been suggested as cutoff for inadequately low dexamethasone (1, 2). This suggestion corresponds well with our observations. In some cases, unexpectedly high plasma dexamethasone levels have been associated with false-negative DST results (3, 14, 15); however, the risk of false-negative DST results could not be assessed in our study.

Several conditions that are common features of Cushing's syndrome, including obesity, diabetes, and depression, may cause physiological hypercortisolism and false-positive DST results (1). Therefore, the association of dexamethasone with cortisol levels may differ between the general population and patients with suspected Cushing's syndrome. We studied Cushing's syndrome suspects, as we aimed to examine how the association between dexamethasone and cortisol levels could be used to improve the DST performance in the evaluation of Cushing's syndrome.

In patients with Cushing's syndrome, dexamethasone and cortisol levels are less likely to be associated, and we excluded patients in whom Cushing's syndrome was confirmed. However, some people with Cushing's syndrome may have posttest plasma cortisol <100 nmol/l (1, 16), which we used as cutoff for insufficient suppression. Therefore, we cannot exclude the possibility that some subjects with undetected Cushing's syndrome may have been included in the analyses. Nonetheless, endogenous Cushing's syndrome is rare (1), and with an expected incidence of endogenous (nonmalignant) Cushing's syndrome of 2.3/million per year (17), we would only expect around two cases to be detected in the referral population of our department during the 2 years of data collection. Therefore, it seems unlikely that any number of patients with Cushing's syndrome included in the analyses could be sufficiently high to substantially attenuate any association between dexamethasone and cortisol levels.

The antibodies of the dexamethasone RIA may have a moderate reactivity with the dexamethasone metabolite 6β-hydroxydexamethasone (average 50% cross-reactivity of 6β-hydroxydexamethasone, 10%) (8), and it is possible that mass spectrometry (18) could provide a more reliable measure of the dexamethasone levels.

In conclusion, 94% of our subjects attained an 0800 h plasma dexamethasone level >5 nmol/l after the 1 mg overnight DST, and in these subjects, dexamethasone levels were not associated with posttest cortisol levels. Our findings suggest that routine comparison of dexamethasone and cortisol levels is not likely to substantially improve the performance of the DST. Nonetheless, dexamethasone measurements may identify subjects with inadequately low dexamethasone levels and may therefore be of value when retesting subjects with possibly false-positive DST results.

## Figures and Tables

**Table 1:** Characteristics of the 53 subjects, given as mean (s.d.) unless otherwise noted.

Characteristic^a^	
Women/men (*n* (%))	36 (68)/17 (32)
Age (years)	56 (14)
Reason for dexamethasone suppression testing (*n* (%))	
Clinical suspicion of Cushing's syndrome	27 (51)
Evaluation of adrenal incidentaloma	26 (49)
Pretest morning plasma cortisol (nmol/l; *n*=48)	550 (196)
Previous surgical treatment for Cushing's disease (*n* (%))	3 (6)
Body mass index (kg/m^2^; *n*=45)	29.6 (5.4)
Systolic/diastolic blood pressure (mmHg; *n*=52)	144 (24)/84 (13)
Previously diagnosed hypertension (*n* (%))	34 (64)
HbA1c (%), median (IQR) (*n*=38)	6.3 (6.0–7.5)
Previously diagnosed diabetes (*n* (%))	18 (34)
Current/former/never smokers (*n* (%) (*n*=50))	17 (34)/5 (10)/28 (56)
Time of posttest blood sampling (median (IQR))	0810 h (0804–0816 h)

IQR, interquartile range.

a For characteristics with incomplete information, the number of subjects with available information is given in parentheses.
